# Deep Brain Stimulation in Parkinson's Disease: Still Effective After More Than 8 Years

**DOI:** 10.1002/mdc3.13040

**Published:** 2020-09-21

**Authors:** Birgitte L.C. Thomsen, Steen R. Jensen, Anders Clausen, Merete Karlsborg, Bo Jespersen, Annemette Løkkegaard

**Affiliations:** ^1^ Department of Neurology Bispebjerg and Frederiksberg University Hospital Copenhagen Denmark; ^2^ Faculty of Health and Medical Science University of Copenhagen Copenhagen Denmark; ^3^ Department of Neurosurgery Rigshospitalet University Hospital Copenhagen Denmark

**Keywords:** Parkinson's disease, deep brain stimulation, long‐term follow‐up, subthalamic nucleus

## Abstract

**Background:**

Deep brain stimulation of the subthalamic nucleus (STN‐DBS) is well established and the most effective treatment for advanced Parkinson's disease (PD). However, little is known of the long‐term effects.

**Objectives:**

The aim of this study was to examine the long‐term effects of STN‐DBS in PD and evaluate the effect of reprogramming after more than 8 years of treatment.

**Methods:**

A total of 82 patients underwent surgery in Copenhagen between 2001 and 2008. Before surgery and at 8 to 15 years follow‐up, the patients were rated with the Unified Parkinson's Disease Rating Scale (UPDRS) with and without stimulation and medicine. Furthermore, at long‐term follow‐up, the patients were offered a systemic reprogramming of the stimulation settings. Data from patients' medical records were collected. The mean (range) age at surgery was 60 (42–78) years, and the duration of disease was 13 (5–25) years. A total of 30 patients completed the long‐term follow‐up.

**Results:**

The mean reduction of the motor UPDRS by medication before surgery was 52%. The improvement of motor UPDRS with stimulation alone compared with motor UPDRS with neither stimulation nor medication was 61% at 1 year and 39% at 8 to 15 years after surgery (before reprogramming). Compared with before surgery, medication was reduced by 55% after 1 year and 44% after 8 to 15 years. After reprogramming, most patients improved.

**Conclusions:**

STN‐DBS remains effective in the long run, with a sustained reduction of medication in the 30 of 82 patients available for long‐term follow‐up. Reprogramming is effective even in the late stages of PD and after many years of treatment.

View Supplementary Video 1

Deep brain stimulation of the subthalamic nucleus (STN‐DBS) is a well‐established treatment of advanced, levodopa‐responsive, fluctuating Parkinson's disease (PD). DBS significantly reduces cardinal motor symptoms and the need for medication,^1^ thereby alleviating adverse effects and fluctuations.^2^ Randomized controlled trials have demonstrated the superiority of DBS over best medical therapy regarding motor outcome and quality of life.^3^, ^4^ However, little is known of the long‐term effects, adverse effects, complications, and prognosis of patients who live with DBS for many years. Until now, only a few studies have investigated the long‐term outcomes (>8 years).^5^, ^6^, ^7^, ^8^, ^9^ These studies showed a sustained effect of stimulation on motor symptoms, although the effect has been described as declining over time. Furthermore, in only a few of these studies the patients' stimulators were turned off at the follow‐up evaluations^6^, ^7^, ^9^ for estimation of the stimulation effect. The lasting effect of treatment in a group of patients with PD with a long disease duration of up to 33 years has not been previously evaluated in this way.The aim of this study was to examine if STN‐DBS treatment is still effective after more than 8 years and in a late stage of PD. Also, the study evaluates the effect of changing the stimulation parameters at a long‐term follow‐up.

## Methods

A total of 82 patients with advanced PD underwent bilateral STN‐DBS surgery at Copenhagen University Hospital between 2001 and 2008. A total of 81 patients were included in this study, as 1 patient did not wish to participate. Of these patients, 62 lived for at least 8 years. A total of 38 patients were still alive when the study was initiated, and 30 patients were included for the long‐term follow‐up examination (Table 1). From the time of surgery, the patients were seen regularly at the movement disorder clinic by the DBS team, with both neurologists and specialist nurses. The patients who were included for a long‐term clinical follow‐up were last seen in the clinic between 1 week and 1 year before. Seven patients were not eligible for clinical follow‐up as a result of severe dementia.

**TABLE 1:** *Patient demography*

Variable	Available for Long‐Term Follow‐Up	Not Available for Long‐Term Follow‐Up	*P* Value
N	30	51	
Sex, female/male, n (%)	11/19 (36.7/63.3)	20/31 (39.2/60.8)	1.0
Age at surgery, y, mean (range)	56.1 (41.9–70.0)	62.5 (47.8–77.6)	0.0002
Age at Parkinson's disease debut, y, mean (range)	44.2 (30–59)	48.4 (36–66)	0.01
Disease duration at surgery, y, mean (range)	11.5 (5–20)	13.9 (5–25)	0.03
Duration of disease at follow‐up, y, mean (range)	23.2 (16.4–32.6)		

The long‐term follow‐up examination group included patients in a clinical long‐term follow‐up examination.

Patients' severity of symptoms was assessed preoperatively and postoperatively after 1 and 8 to 15 years with the Unified Parkinson's Disease Rating Scale (UPDRS). Preoperatively, patients were evaluated in a self‐reported *on* condition following their usual medical regimen and in a defined *off* condition in the morning 12 hours after withdrawal of antiparkinsonian medication.

At the 1‐year follow‐up, patients were assessed in 3 conditions: in the ON/*on* condition with their normal stimulation and medication regimen, in the ON/*off* condition with stimulation but withdrawal of medication for 12 hours, and OFF/*off* with no stimulation and no medication for 12 hours.

The long‐term follow‐up 8 to 15 years after surgery was carried out in the same way as the 1‐year follow‐up, except that the stimulation was turned off only for as long as the patient could manage and no longer than 3 hours.

At the long‐term follow‐up, patients were offered a systematic testing of the contact points to explore whether DBS settings could be optimized. This evaluation took place during a short hospitalization. The testing proceeded by a systematic protocol (Appendix S1). After 2 to 5 weeks with the new setting, a reevaluation was performed.

Clinical medical records were reviewed from the time of the preoperative examination to the time of the long‐term follow‐up (July 1, 2017) or the death of the patient. Adverse effects were assessed clinically and registered to derive from medication or stimulation based on previous experience and whether they changed with surgery or when regulating medication or stimulation dose.^10^ Neuropsychological testing was performed before surgery to exclude dementia. From the retrospective curation of patient files, it was noted if patients were given a diagnosis of dementia either based on a neuropsychological examination or based on the initiation of treatment with an acetylcholine‐esterase inhibitor. Antiparkinsonian drugs were converted into levodopa equivalent daily doses (LEDD).^11^ Some patients were prescribed a small dosage of pro re nata medications, always less than 5% of the total LEDD. The exact dosages used could not be curated from the medical records. Based on clinical practice and as a pragmatic solution it was decided to use 50% of the dosage in the evaluation.

### Surgical Procedure

All patients fulfilled the inclusion criteria for DBS surgery based on the original recommendations from the Core Assessment Program for Surgical Interventional Therapies (CAPSIT) protocol:^12^ a diagnosis of levodopa‐responsive PD for a duration of at least 5 years, motor fluctuations and levodopa‐induced dyskinesia despite optimized medical therapy, Hoehn and Yahr scale ≥ 3.5, and no dementia (Mini Mental State Examination > 24). Exclusion criteria were significant psychiatric disorders and contraindications to surgery. Patients were bilaterally implanted with a quadripolar DBS electrode (Model Soletra 7426 or Kinetra 7428, Medtronic, Dublin, Ireland) after 12 hours discontinuation of anti‐parkinsonian medication (over‐night). Surgery was performed under local anesthesia using magnetic resonance imaging–guided stereotactic surgery. The frame used was CRW Radioonics and the software used to plan the target was Radionics Stereoplan (Burlington, MA, USA) and later Brainlab iPlan (Munich, Germany). An intraoperative single‐track microrecording was used to secure the target. Verification of the electrode locations was made using macrostimulation, clinical testing, and X‐ray. Following lead implantation, the pulse generator and the subcutaneous extension wire were implanted under general anesthesia. The electrodes used were Medtronic lead model 3389 (small electrode spacing) (Table S1 in Appendix S1).

### Statistical Analysis

The *t* and chi‐squared tests were used to compare patient demography (Table 1). Longitudinal UPDRS data was evaluated using mixed model analysis that allow for missing data. UPDRS subscores were examined individually for tremor (item 20 + 21), rigidity (item 22), bradykinesia (item 23–26 + 31), gait (item 29), and postural instability (item 30). UPDRS III was transformed with the natural logarithm to achieve the best normal distribution. A Wilcoxon signed‐rank test was used to compare UPDRS data after reprogramming. Mann‐Whitney *U* and chi‐squared tests were used to compare patients who agreed and disagreed to undergo reprogramming. Medicine intake was analyzed using a paired‐sample *t* test. Mixed model analysis was used to analyze voltage due to missing data, and Wilcoxon signed‐ranked test was used on frequency and pulse width due to nonnormal distribution. A competing risk model was used to analyze dementia. A Kaplan‐Meier estimator was used to analyze survival, and the McNemar test was used to analyze complications and adverse effects. A 2‐sided probability (*P*) value ≤ 0.05 was considered significant.

**FIG. 1:**
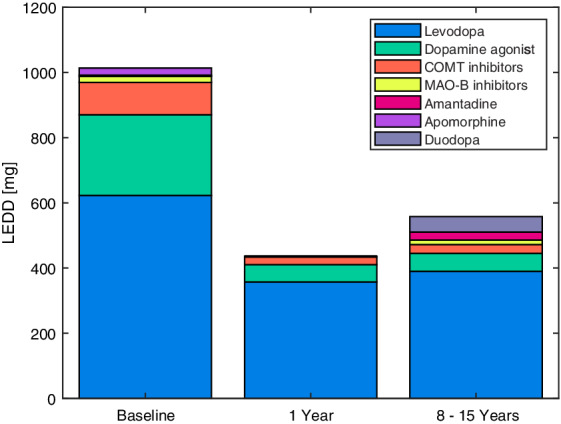
The mean LEDD in milligrams divided into levodopa, dopamine agonists, COMT inhibitors, MAO‐B inhibitors, amantadine, apomorphine, and Duodopa at baseline (N = 81), 1 year (N = 78), and 8 to 15 years after surgery (N = 62). COMT, catechol‐O‐methyltransferase; LEDD, levodopa equivalent daily dose; MAO‐B, monoamine oxidase B.

Data were analyzed using Statistical Analysis Software (SAS Institute, Cary, NC, USA). MATLAB (MathWorks, Natick, MA, USA) was used to generate Figure 1. The results will be stated as mean (standard deviation) unless differently explained.

## Results

Time between surgery and the 1‐year follow‐up was 1.1 (range, 0.94–1.7) years. Mean time to long‐term follow‐up was 12 (range, 8.5–15) years.

### Effect on Motor Symptoms

Before surgery, the motor symptoms (UPDRS III) were significantly reduced by 52% by medication (*P* < 0.0001). STN‐DBS significantly reduced UPDRS III by 61% when comparing the OFF/*off* and the ON/*off* conditions 1 year after surgery (*P* < 0.0001) and significantly by 39% at the long‐term follow‐up 8 to 15 years after surgery (*P* < 0.0001).

Medicine significantly reduced motor symptoms further when added to the DBS treatment. The effect of both STN‐DBS and medicine (ON/*on*) was a reduction of motor symptoms by 69% 1 year after surgery (*P* < 0.0001), and by 51% at the long‐term follow‐up compared with the OFF/*off* condition (*P* < 0.0001) (Table 2).

**TABLE 2:** *Treatment effect*

UPDRS motor score	Time							
	Baseline	Baseline	1 Year	1 Year	1 Year	8–15 Years	8–15 Years	8–15 Years
	OFF Treatment	ON Treatment	OFF Treatment	ON/*Off* Treatment	ON/*On* Treatment	OFF Treatment	ON/*Off* Treatment	ON/*On* Treatment
Total	43.8 (9.1)	21.1 (8.8)^a^	48.5 (8.4)	19.1 (8.7)^a^	14.9 (8.5)^a^ ^,^ ^b^	57.9 (5.2)	35.3 (5.8)^a^	28.4 (5.9)^a^ ^,^ ^b^
Tremor	6.0 (5.3)	1.6 (2.9)^a^	7.7 (5.2)	1.6 (1.8)^a^	0.98 (1.5)^a^ ^,^ ^c^	6.6 (4.1)	2.4 (3.2)^a^	1.1 (1.4)^a^ ^,^ ^c^
Rigidity	9.7 (2.8)	5.6 (2.6)^a^	9.7 (3.1)	4.0 (2.4)^a^	3.3 (2.4)^a^ ^,^ ^c^	12.2 (4.0)	7.2 (4.2)^a^	5.3 (3.5)^a^ ^,^ ^b^
Bradykinesia	19.8 (5.5)	10.9 (5.4)^a^	22.8 (5.8)	10.6 (5.5)^a^	8.4 (5.2)^a^ ^,^ ^b^	27.2 (3.6)	18.6 (4.4)^a^	14.5 (4.3)^a^ ^,^ ^b^
Gait	1.9 (1.0)	0.88 (0.81)^a^	1.8 (1.1)	0.88 (0.86)^a^	0.68 (0.73)^a^ ^,^ ^c^	2.5 (1.3)	1.7 (1.2)^d^	1.6 (1.1)^d^
Postural instability	1.6 (0.97)	0.90 (0.75)^a^	1.5 (1.0)	0.76 (0.87)^a^	0.66 (0.71)^a^	2.6 (0.99)	1.9 (1.2)^a^	1.7 (1.1)^a^

^a^ *P* value <0.0001 compared with OFF at the same time.

^b^ *P* value <0.0001 compared with ON/*off* at the same time.

^c^ *P* value <0.05 compared with ON/*off* at the same time.

^d^ *P* value <0.05 compared with OFF at the same time.

Data are presented as mean (standard deviation).

UPDRS, Unified Parkinson's Disease Rating Scale.

When comparing the OFF/*off* and the ON/*off* scores at the 1‐year follow‐up, stimulation significantly reduced tremor by 80% (*P* < 0.0001), rigidity by 58% (*P* < 0.0001), and bradykinesia by 53% (*P* < 0.0001); improved gait by 52% (*P* < 0.0001); and reduced postural instability by 50% (*P* < 0.0001). At the long‐term follow‐up, STN‐DBS significantly reduced tremor by 64% (*P* < 0.0001), rigidity by 41% (*P* < 0.0001), and bradykinesia by 32% (*P* < 0.0001); improved gait by 30% (*P* = 0.0009); and reduced postural instability by 29% (*P* < 0.0001).

### Effect on Active Daily Living

The effect of treatment on activity of daily living according to the UPDRS II OFF compared with UPDRS II ON was a significant reduction by 37% before surgery (*P* < 0.0001). At 1‐year after surgery, the effect of stimulation and medicine (ON/*on*) was a significant reduction of 59% (*P* < 0.0001) and 8 to 15 years after surgery by 33% (*P* < 0.0001).

### Alleviation of Adverse Effects Attributed to Medication

UPDRS IV was significantly reduced by 78% between baseline and 1 year after surgery (*P* < 0.0001), and 70% between baseline and 8 to 15 years after surgery (*P* < 0.0001). Significantly fewer patients experienced dyskinesia, dystonia, fluctuations, *off* periods, and hallucinations attributed to medication 1 year after surgery compared with baseline. The effect remained at the long‐term follow‐up for dyskinesia, fluctuations, and *off* periods. Significantly more patients suffered from hallucinations at the long‐term follow‐up compared with before surgery and 1 year after surgery. Significantly more patients suffered from medicine‐induced dystonia, fluctuations, *off* periods, vivid dreams, and hallucinations at the long‐term follow‐up compared with 1 year after surgery (Table 3).

**TABLE 3:** Complications and Adverse Effects

Complication/Adverse Effect	Presurgery	Postsurgery	1 Year	8–15 Years
Number of patients	81	81	78	62
Related to surgery, n (%)				
Hemorrhage		1 (1.2)		
Air at the frontal lobe		2 (2.5)		
Infection around the battery and/or cables		11 (13.6)		
Suspected infection around the battery and/or cables		7 (8.6)		
Misplaced electrode(s)		6 (7.4)		
Skin defect		4 (4.9)		
Lead migration		0 (0)		
Confusion		8 (9.9)		
Manic episode		2 (2.5)		
Depressive episode		7 (8.6)		
Fracture attributed to fall		1 (1.2)		
Stimulation and medicine, n (%)				
Dyskinesia	71 (87.7)		25 (32.1)	29 (46.8)
Fluctuations	73 (90.1)		10 (12.8)	14 (22.6)
*Off* periods	77 (95. 1)		19 (24.4)	21 (33.9)
Stimulation, n (%)				
Hardware failure		0 (0)	2 (2.6)	0 (0)
Speech difficulty		14 (17.3)	13 (16.7)	15 (24.2)
Postural instability		5 (6.2)	3 (3.8)	2 (3.2)
Falling		2 (2.5)	2 (2.6)	0 (0)
Dystonia		9 (11.1)	11 (14.1)	10 (16.1)
Psychosis		0 (0)	0 (0)	0 (0)
Mania		2 (2.5)	1 (1.3)	0 (0)
Problematic weight gain		1 (1.2)	5 (6.4)	0 (0)
Impulse control disorder		0 (0)	0 (0)	0 (0)
Double sight		1 (1.2)	1 (1.3)	1 (1.6)
Sensory complaints		2 (2.5)	1 (1.3)	0 (0)
Eyelid apraxia or blepharospasms		1 (1.2)	1 (1.3)	1 (1.6)
Apathy		2 (2.5)	4 (5.5)	3 (4.8)
Medicine, n (%)				
Dyskinesia	71 (87.7)		25 (32.1)	29 (46.8)
Dystonia	28 (34.6)		5 (6.4)	15 (24.2)
Fluctuations	73 (90.1)		10 (12.8)	14 (22.6)
*Off* periods	77 (95.1)		19 (24.4)	21 (33.9)
Impulse control disorder	1 (1.2)		0 (0)	2 (3.2)
Nightmares	6 (7.4)		1 (1.3)	5 (8.1)
Vivid dreams	4 (5.0)		2 (2.6)	10 (16.1)
Hallucinations	12 (14.8)		2 (2.6)	24 (38.7)
Mania	2 (2.5)		2 (2.6)	2 (3.2)

Complications of deep brain stimulation surgery and adverse effects attributed to deep brain stimulation or medicine were registered according to the medical records presurgery and 1 year and 8 to 15 years after surgery. Medically induced *on* and *off* phase dystonia are reported together.

### Optimization of DBS Settings

Optimization of the stimulation setting was protocoled within the first year after surgery in our center. After that, a more thorough systematic evaluation was only performed if patients were experiencing changes in effect or emerging side effects. As a part of this long‐term follow‐up, all included patients were offered a systematic testing with a possible reprogramming of DBS settings regardless of symptoms. Of 30 patients, 25 agreed to the testing of different DBS settings. The 5 patients who did not want a test of the setting had similar age, duration of PD, duration of DBS treatment, UPDRS total/motor in OFF/*off* or ON/*on* stages, DBS effect, and LEDD. None of these 5 patients had dementia, whereas 13/25 patients who agreed to testing were registered as demented (*P* = 0.053). In 20 of 25 (80%) tested patients, the setting was adjusted (Table S1 in Appendix S1). Two patients were not interested in the reevaluation with UPDRS. The mean ON/*on* UPDRS III was 30.7 (10.9) before reprogramming and 27.7 (12.2) after reprogramming, a reduction of 3.0 (10%) (*P* = 0.004). Rigidity significantly improved by 0.89 (17%) (*P* = 0.02), whereas tremor improved by 0.28 (29%), bradykinesia 1.39 (9.4%), gait 0.11 (6.7%), and postural instability 0.056 (3.2%), however, not significantly. A total of 15 (83%) patients improved, and 3 (17%) patients worsened. For the patients who improved, the mean improvement of UPDRS III was 4.1 (13%) (*P* < 0.0001).

The adjustment of DBS settings significantly improved total ON/*on* UPDRS from 59.8 (21.3) before reprogramming to 55.1 (23.5) after reprogramming. This corresponds to a reduction by 4.7 (7.8%) (*P* = 0.007). In 16 patients, total UPDRS was performed. A total of 13 (81%) patients improved 6.6 (11%) after stimulator setting changes (*P* = 0.0002). Two (13%) patients had worsened, and 1 patient (6.3%) was unchanged.

### Medication

The mean intake of antiparkinsonian medication in LEDD was 1014 (473) mg before surgery, 439 (294) mg after 1 year, and 559 (431) mg after 8 to 15 years (Fig. 1). The percentages of dopamine agonists and controlled‐release medication were, respectively, 24.4% and 8.8% before surgery, 12.1% and 9.1% 1 year after surgery, and 9.9% and 4.6% 8 to 15 years after surgery. The total intake of dopaminergic medication was significantly reduced 1 year after surgery by 55% compared with baseline (*P* < 0.0001) and by 44% at the long‐term follow‐up compared with baseline (*P* < 0.0001). Two patients were both treated with STN‐DBS and a Duodopa pump (Abbvie, North Chicago, IL, USA) at the long‐term follow‐up as a result of decreased effectiveness of the STN‐DBS and adverse effects to oral medications.

### Stimulation

According to pre‐operative observations, all patients had asymmetric parkinsonism, with more severe motor symptoms on one side, and the corresponding dominant hemisphere was registered. Stimulation was commenced within the first week after surgery and in most patients on the second day. A standard protocol was applied (Appendix S1).

The mean voltage on the dominant side increased over time from 2.3 mV (*P* < 0.0001) postoperatively to 3.0 mV at the 1‐year follow‐up and 3.2 mV at 8 to 15 years follow‐up (*P* = 0.06). On the nondominant side, voltage increased from 2.2 mV postoperatively to 2.8 at the 1‐year follow‐up (*P* < 0.0001) and to 3.1 mV at 8 to 15 years follow‐up (*P* = 0.002). During the first year, frequency was significantly increased bilaterally (*P* < 0.001), and pulse width increased significantly on the dominant side (*P* = 0.03). Between the 1‐year follow‐up and 8 to 15 years follow‐up, the frequency and pulse width did not change significantly (Table S4 in Appendix S1).

A total of 68 patients had 1 or more battery changes, 50 patients had 2 battery changes, 22 patients 3 battery changes, 11 patients had 4 battery changes, and 3 patients had 5 battery changes (Table S2 in Appendix S1).

### Dementia and Mortality

**FIG. 2:**
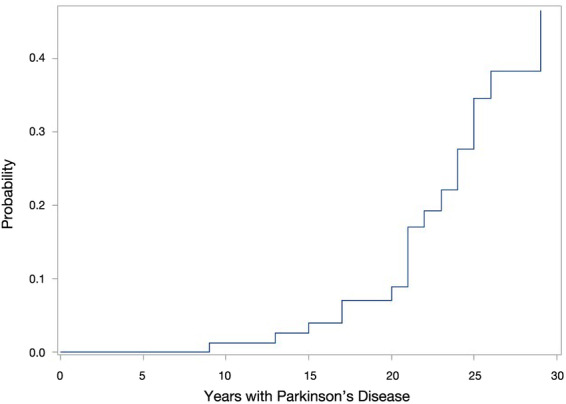
Survival analysis of dementia with death as competing risk in accordance with years of Parkinson's disease.

Of 81 patients, 43 (53%) were diagnosed with dementia before death or before the long‐term follow‐up. The mean age for diagnosis of dementia was 67.0 (5.9) years with a mean disease duration of 20.5 (5.9) years. The time between surgery and the occurrence of dementia in this group was 5.8 (3.3) years. The time from the diagnosis of dementia to death was 4.3 (2.7) years (Fig. 2).

**FIG. 3:**
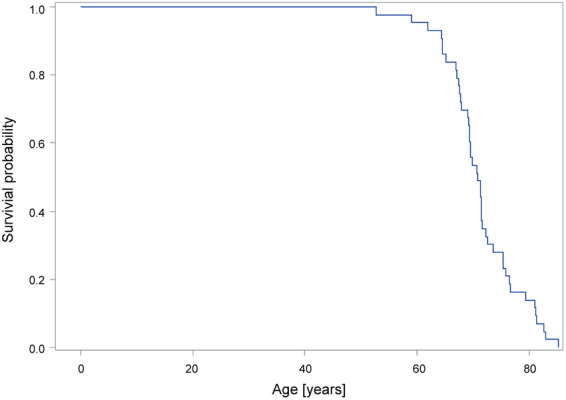
Survival analysis in accordance with age. Causes of death are old age (n = 14), pneumonia (n = 11), cancer (n = 7), stroke (n = 3), myocardial infarct (n = 1), lung embolism (n = 1), application of percutaneous endoscopic gastrostomy tube (n = 1), suicide (n = 1), unknown (n = 4).

Of 81 patients, 43 (53%) died before the long‐term follow‐up. The mean age at death was 71.3 (6.5) (range 52.7–85.2) years. The mean duration of PD at the time of death was 22.2 (6.3) (range 10.7–38.9) years, and the mean time span between surgery and death was 8.1 (3.5) (range 0.58–13.5) years. According to a survival analysis, the estimated median (95% confidence interval) age at death was 70.9 (69.1–71.6) years (Fig. 3).

### Complications and Adverse Effects

A total of 30 patients (37%) had complications within 30 days after surgery. One patient (1.2%) suffered from hemorrhage during surgery. Two patients (2.5%) had pneumocephalus. The patient who suffered from hemorrhage and 1 of the patients who suffered from pneumocephalus (2.5%) had persisting hemiparesis after surgery (Table 3).A total of 14 patients (17%) experienced complications attributed to battery replacements. At the first replacement 4%, at the second 12%, and from the third replacement ≥25% of patients suffered complications (Table S2 in Appendix S1).

One patient committed suicide 1.2 years after surgery. The patient started antidepressant medication 3 months after surgery. He was regularly followed, and no hallucinations or psychoses were reported.

None of the adverse effects attributed to DBS became significantly more frequent over time (Table 3).

## Discussion

This study adds important evidence regarding long‐term outcomes of STN‐DBS in PD. Of 81 patients, 30 were available for long‐term follow‐up examination. We demonstrated that STN‐DBS has a lasting albeit somewhat waning effect on motor symptoms and allows for a marked reduction of dopaminergic replacement therapy even after a mean follow‐up time of 12 years after surgery and a mean disease duration of 23 years. A slight decrease of the DBS effect seen over the years was expected based on previous studies.^6^, ^7^, ^9^

We found a lasting effect of stimulation, reducing motor symptoms by 39% in accordance with other long‐term studies (24%–34%).^6^, ^7^, ^9^ At the 8 to 15 years follow‐up, stimulation significantly reduced all cardinal symptoms: tremor, bradykinesia, rigidity, postural instability, and gait problems. Also, a significant improvement of activity of daily living functions was demonstrated. The addition of medicine resulted in a further reduction of motor symptoms both 1 year after surgery and at long‐term follow‐up.

Furthermore, we found STN‐DBS to be a safe treatment, even with long‐term follow‐up. The alleviation of adverse effects as a result of medication was maintained probably as a result of the lasting reduction of medication, which was 44% at the long‐term follow‐up compared with baseline. Other long‐term follow‐up studies reported medicine reductions of 23% to 49% after 6 months^3^, ^4^ and 36% to 60% after >8 years.^5^, ^7^, ^8^ This diversity is probably primarily reflecting different treatment strategies as no formal guidelines exist.^13^

### Systematic Reprogramming of DBS Settings

This study demonstrated for the first time that a systematic evaluation and optimization of stimulator settings is valuable and may result in significant improvements 8 to 15 years after surgery. These patients had been followed in our out‐patient clinic on a regular basis. Nevertheless, we found an effect on the short‐term outcome, as a positive effect was seen on all UPDRS subitems after 2 to 5 weeks.

An effect of systematic reprogramming has previously been reported also by Moro and colleagues^14^ in a younger group 3.5 years after surgery. Of the patients, 89% received a new setting, and a sustained effect was found in more than half of the patients after up to 14 months. In contrast, our group was older, and we demonstrated an effect of reprogramming in a group of more advanced patients with a mean stimulation period of 12 years. The results have changed the treatment strategy in our clinic, where reprogramming has been implemented more regularly as of today and also after a long treatment period.

### Dementia and Mortality

Of the patients, 53% were diagnosed with dementia before the time of death or before the time of the long‐term follow‐up. The mean age at the time of the diagnosis of dementia was 67.0 years with a disease duration of 20.5 years. This was not more than expected. According to the Sydney Multicenter Study, where a prospective cohort of patients with PD was followed for 20 years, 83% of patients were diagnosed with dementia.^15^

Likewise, the mortality was not higher than expected as the expected median (95% confidence interval) age of death was 70.9 (69.1–71.6) years. The median expected life expectancy for the whole patient group was calculated in proportions to the age at PD onset to be 70.4 years.^16^ A study found a lower mortality after STN‐DBS surgery when comparing patients who underwent surgery with those who were offered surgery but declined. This was mainly attributed to the lower prevalence of death from pneumonia.^17^ The authors concluded that STN‐DBS decreases mortality in PD as a result of better mobility.

### Safety

In accordance with previous reports, we found that STN‐DBS is a safe neurosurgical procedure. Of 81 patients, 1 (1.2%) had a hemorrhage during surgery, which is consistent with the incidence previously reported.^18^ No deaths occurred as a result of surgery. One patient in our group (1.2%) committed suicide 1.2 years after surgery. An increased risk of suicide has been suggested with STN‐DBS with an incidence between 0.16% and 0.45%;^19^, ^20^ however, no clear connection has been found.^21^, ^22^

Battery replacements were relatively safe the first and second times, but the risk of complications increased significantly from the third time with a prevalence of ≥25%. In a long‐term study, 4 of 79 patients (5.1%) suffered from infection as a result of battery changes.^23^ Because of the high risk of complications after several battery changes, it is relevant to consider implanting only rechargeable batteries in younger patients.

We found that 37% of patients had adverse effects attributed to stimulation, which is comparable with previous studies. The most common adverse effects attributed to stimulation reported in our group were speech difficulties and dystonia. Of the patients, 17% experienced speech difficulties attributed to stimulation at the 1‐year follow‐up and 24% at the 8 to 15 years follow‐up. The reported prevalence of speech difficulties as an adverse effect attributed to STN‐DBS varies widely from 4.8%^24^ to 70%.^5^ Dystonia was reported in 14% of the patients at the 1‐year follow‐up and 16% at the 8 to 15 years follow‐up. Another long‐term follow‐up study found that 13% of the patients had persisting limb dystonia after 8 years.

^5^

STN‐DBS is generally believed to impair balance,^25^, ^26^ and one randomized controlled study comparing STN‐DBS and internal globus pallidus (GPi)‐DBS with best medical treatment found that falls and gait disturbances were significantly more frequent in patients treated with STN‐DBS and GPi‐DBS for 4 to 6 months compared with patients receiving best medical therapy.^4^ However, we found an improvement of postural stability in the OFF/*on* state compared with the OFF/*off* state at baseline, 1 year follow‐up, and long‐term follow‐up. In addition, postural stability and gait were improved 1 year after surgery in the ON/*on* state compared with baseline ON (Table 2). Similarly, other long‐term follow‐up studies have found a significant improvement of gait, postural instability, and falls.^27^, ^28^ When postural stability in the ON state at long‐term follow‐up was compared with baseline ON, a significant impairment was seen that could be expected as a result of the progression of disease.

### Limitations

This study has limitations based on the retrospective nature and the long follow‐up time. A more unbiased approach could have been sought with blinded video ratings of the UPDRS.^7^

A possible source of error is the practical *off* state of the 12‐hour pause in medicine. This means that some influence of antiparkinsonian medication and stimulation must be expected. In our study, the *on* state was defined as the state the patient reported as a good *on* state, most often but not always in the morning and not after a predefined intake of medicine, as done by many other studies.^3^, ^4^, ^5^, ^6^, ^7^, ^8^, ^9^ Therefore, our *on* UPDRS scores might be lower compared with other studies, which might result in a relative underestimation of the treatment effect.

As this is a long‐term follow‐up study partly based on retrospective data collection, some data are missing. Some of the UPDRS and stimulation setting charts were physically lost and not saved electronically. The possible error caused by data missing at random was corrected using mixed model analysis. Data not missing at random were handled by excluding the patients in the analysis.

Reprogramming of the STN‐DBS settings were tested in 18 patients and showed a significant mean improvement slightly larger than what has been described as the minimal clinically relevant change. This needs to be tested in more patients, preferably in a blinded fashion.

As adverse effects were registered retrospectively and not systematically, we depended on what had been noted in the clinical files. Whether an adverse effect was attributed to medication, stimulation, or both could not be differentiated with certainty and was based on a clinical estimate.^29^

### Conclusions

This study significantly extends the knowledge on the long‐term effects of STN‐DBS in a consecutive group of 81 patients with PD and followed during a time period of up to 15 years. At the time of follow‐up, 30 patients were available for a long‐term examination. The data obtained from the study show that STN‐DBS is an efficient treatment of advanced PD both short and long term after a mean of 12 years with STN‐DBS and a disease duration of up to 33 years.

We have, for the first time, demonstrated a significant effect of stimulation reprogramming more than 8 years after surgery in otherwise well‐treated patients. The complications attributed to battery replacements increased steeply after the second change, leading to the conclusion that rechargeable batteries should always be considered as a first option.

## Author Roles

(1) Research Project: A. Conception, B. Organization, C. Execution; (2) Statistical Analysis: A. Design, B. Execution, C. Review and Critique; (3) Manuscript Preparation: A. Writing of the First Draft, B. Review and Critique.

B.L.C.T.: 1C, 2A, 2B, 3A

S.R.J.: 1B, 1C, 3B

A.C.: 1B, 1C, 3B

M.K.: 1A, 1B, 1C, 3B

B.J.: 1C, 3B

A.L.: 1A, 1B, 1C, 2A, 2C, 3B

## Disclosures

**Ethical Compliance Statement**: The Danish National Committee has approved the study (reference no.: H‐15007736). The authors confirm that informed consent was obtained for all participants who were alive at the time of the study. Patient consent was not required from the patients who had died before the study was performed. We confirm that we have read the Journal's position on issues involved in ethical publication and affirm that this work is consistent with those guidelines.

**Funding Sources and Conflicts of Interest**: Funding source for study was The Danish Parkinson's Association. B.L.C.T., S.R.J., A.C., M.K., B.J. and A.L. report no conflicts of interest.

**Financial Disclosures for the Previous 12 Months**: B.L.C.T. reports a research grant from The Danish Parkinson's Association, funding of congress registration and travel grant from the Movement Disorders Society, and funding of a travel grant from The Oticon Foundation. S.R.J. reports a research grant from The Danish Parkinson's Association. A.C. reports a research grant from The Danish Parkinson's Association and funding of a meeting from Medtronic. M.K. reports lecture honorarium from AbbVie A/S. B.J. has no disclosures to report. A.L. reports a research grant from The Danish Parkinson's Association, funding of a meeting from Medtronic, and lecture honorarium and funding of congress registration from AbbVie A/S.

## Supporting information


**Appendix S1.** The supplementary material includes the following: (1) the protocol for systematic programming, (2) changes made in systematic reprogramming, (3) information about when battery replacements were made, and (4) an overview of complications attributed to surgery. Furthermore, tables show (1) the Unified Parkinson's Disease Rating Scale and stimulation settings before and after reprogramming at the long‐term follow‐up for each patient, (2) complications to battery replacements, and (3) the stimulation settings over time.

**Video S1.** A patient with 11 years of Parkinson's disease at 60 years of age before subthalamic deep brain stimulation and at long‐term follow‐up 10 years later. First, the patient is seen at baseline in the *off* state (no medication for 12 hours; Unified Parkinson's Disease Rating Scale 3 = 46) and then in the *on* state (with usual medicine regimen; levodopa equivalent daily dose = 2197 mg, Unified Parkinson's Disease Rating Scale 3 = 10). Then he is seen 10 years later in the *off*/OFF state (no medication for 12 hours and stimulation turned off for 3 hours; Unified Parkinson's Disease Rating Scale 3 = 71) and in the *on*/ON state (with the usual medicine regimen and stimulation setting; levodopa equivalent daily dose = 1064 mg, Unified Parkinson's Disease Rating Scale 3 = 30).
