# Supplementary material for: Deep Brain Stimulation in Parkinson's Disease: Still Effective After More Than 8 Years
Source: Mov Disord Clin Pract. 2020 Sep 21;7(7):788–96. doi: 10.1002/mdc3.13040 (PMC7534016; doi:10.1002/mdc3.13040)
Supplement: Supplementary file 1 — Appendix S1. The supplementary material includes the following: (1) the protocol for systematic programming, (2) changes made in systematic reprogramming, (3) information about when battery replacements were made, and (4) an overview of complications attributed to surgery. Furthermore, tables show (1) the Unified Parkinson's Disease Rating Scale and stimulation settings before and after reprogramming at the long‐term follow‐up for each patient, (2) complications to battery replacements, and (3) the stimulation settings over time. [file MDC3-7-788-s001.docx]

Supplementary Material

Protocol for Systematic Programming

The following protocol was both used to program the DBS settings after surgery and to examine the effect of systematic reprogramming at the long-term follow-up.

The testing proceeded as follows: 1) Motor-UPDRS score. 2) The battery of the left hemisphere was switched off for up to one hour. 3) Each electrode point was tested starting from the more caudal to the more cranial. A monopolar configuration was used with the pulse width and frequency set to 60 ms and 130 Hz. Every 0.2 second the voltage was increased with 0.1 mV until it reached 2.0 mV. If no adverse effects were seen after one minute, the voltage was further turned up in intervals of up to 0.5 mV until 4.0 mV was reached or adverse effects occurred. After every 1 mV increment, there was a break of one minute. At the voltage of 4.0 mV, or right before adverse effects occurred there was a pause of 15 minutes. After 15 minutes the effect on tremor, bradykinesia, and rigidity was examined using the corresponding UPDRS subitems. Lastly, the impedance was measured. 4) The battery to the right hemisphere was switched off and the protocol was repeated.

At the long-term follow-up, the new settings were evaluated with a new UPDRS two to five weeks later.

Changes made in systematic reprogramming

In 14 patients the contact points were changed; in 18 patients the voltage was changed and in nine patients frequency was changed.

Battery Changes

The time from the first implanted battery to the first battery change was 4.5 [0.99] years (n=68), 3.8 [0.96] at the second battery change (n=50), 3.3 [1.1] at the third battery (n=22), 2.5 [1.1] at the fourth battery change (n=11), and 2.1 [0.49] years at the fifth battery change (n=3).

Complications

Three patients (3.7 %) had an infection around the battery and/or cables after surgery which necessitated reoperation. In two of these (2.5 %), the equipment was removed permanently. Eight patients (9.9 %) were treated with antibiotics due to infection. Five patients (6.2 %) had one electrode misplaced and one patient (1.2 %) had both electrodes misplaced. Electrode placement was not systematically examined. Misplaced electrodes were discovered due to lack of effectiveness of the stimulation (3 patients) or due to double sight (3 patients). Five of the patients underwent reoperation. Two patients had one electrode replaced and one had both electrodes drawn back a few millimeters. Two patients had their misplaced electrode withdrawn and new ones inserted. One of these had a lead breakage during reoperation.

**Supplementary Table 1.** Reprogramming

| Time | Baseline | Baseline | Baseline | After | After | After |
| --- | --- | --- | --- | --- | --- | --- |
| Patient | UPDRS 2 ON/ON | UPDRS 3 ON/ON | Stimulation setting | UPDRS 2 ON/ON | UPDRS 3 ON/ON | Stimulation setting |
| 1 | 18 | 33 | R: (00-0+)3.3,180,60  L: (--00+)3.7,180,60 | n/a | 34 | R: (00--+)3.0,180,60  L: (0--0+)3.7,180,60 |
| 2 | 19 | 49 | R: (0--0+)3.4,130,60  L: (--00+)3.4,130,60 | 19 | 48 | R: (0--0+)3.5,130,60  L: (00--+)3.5,130,60 |
| 3 | 16 | 21 | R: (0--0+)2.7,150,60  L: (00--+)3.3,150,60 | 15 | 16 | R: (0--0+)3.0,130,60  L: (00--+)3.6,130,60 |
| 4 | 16 | 20 | R: (0--0+)4.1,130,60  L: (0--0+)3.9,130,60 | 16 | 18 | R: (0--0+)3.8,130,60  L: (00--0+)3.5,130,60 |
| 5 | 10 | 18 | R: (00-0+)3.6,160,60  L: (0--0+)3.9,160,90 | n/a | 17 | R: (00-0+)3.8,160,60  L: (0--0+)4.2,160,60 |
| 6 | 33 | 30 | R: (0---+)2.8,130,60  L: (0--+0)3.8,130,60 | 28 | 23 | R: (0--+0)4.0,130,60  L: (0--+0)4.5,130,60 |
| 7 | 22 | 35 | R: (00--+)2.4,170,60  L: (0--0+)3.5,170,60 | 20 | 34 | R: (000-+)4.2,130,60  L: (0-00+)4.2,130,60 |
| 8 | 37 | 42 | R: (000-+)2.2,130,60  L: (-000+)3.0,130,60 | 36 | 45 | R: (0--0+)3.2,160,60  L: (000-+)2.1,160,60 |
| 9 | 8 | 13 | R: (0+--0)3.0,130,60  L: (0--0+)3.5,130,60 | 5 | 5 | R: (0+--0)3.0,130,60  L: (00--+)3.5,130,60 |
| 10 | 12 | 25 | R: (00-0+)2.3,160,60  L: (0-0-+)3.2,160,60 | 10 | 19 | R: (00--+)2.8,130,60  L: (00--+)3.5,130,60 |
| 11 | 18 | 40 | R: (00--+)3.4,130,60  L: (00--+)3.1,130,60 | 18 | 38 | R: (00--+)3.6,160,60  L: (00--+)3.3,160,60 |
| 12 | 19 | 39 | R: (00-0+)2.8,130,60  L: (00--+)3.4,130,60 | 17 | 36 | R: (0+--0)3.3,130,60  L: (00--+)3.4,130,60 |
| 13 | 24 | 32 | R: (00-0+)2.1,160,60  L: (00-0+)2.4,160,60 | 21 | 26 | R: (00-0+)2.5,130,60  L: (00-0+)2.6,130,60 |
| 14 | 17 | 30 | R: (0--0+)3.0,150,60  L: (00--+)3.0,150,60 | 13 | 21 | R: (0---+)3.0,150,60  L: (00--+)3.0,130,60 |
| 15 | 6 | 13 | R: (0-00+)2.1,125,60  L: (000+)5.1,125,60/  (0+0-0)4.0,125,60) | 4 | 10 | R: (0-00+)2.1,125,60  L: (000-+)5.1,125,60/  (0+-00)3.0,125,60 |
| 16 | 16 | 23 | R: (0+--0)3.8,150,60  L: (00-0+)3.3,150,60 | 20 | 26 | R: (00-0+)3.3,130,60  L: (00-0+)3.3,130,60 |
| 17 | 30 | 45 | R: (+-000)4.1,180,60  L: (0+-00)2.2,180,60 | 26 | 43 | R: (+-000)4.1,130,60  L: (0+-00)2.2,130,60 |
| 18 | 27 | 45 | R: (--+00)3.7,190,60  L: (0--+0)3.7,190,60 | 26 | 40 | R: (--+00)3.9,190,60  L: (0+-00)3.0,190,90 |

Legend: UPDRS 2 and 3 ON/ON as well as Stimulation Setting Before and After Reprogramming in Each Patient. R: Right Hemisphere Electrode, L: Left Hemisphere Electrode. Interleaving Settings are Stated as: Setting 1/Setting 2.

**Supplementary Table 2.** Complications to Battery Replacements.

| Number of Battery Replacement | 1^st^ | 2^nd^ | 3^rd^ | 4^th^ | 5^th^ |
| --- | --- | --- | --- | --- | --- |
| N | 68 | 49 | 23 | 13 | 4 |
| Any Complication(s) | 2 (2.9 %) | 6 (12.2 %) | 8 (34.8 %) | 4 (30.8 %) | 1 (25.0 %) |
| Infection | 2 (2.9 %) | 3 (6.1 %) | 6 (26.1 %) | 2 (15.4 %) | 0 |
| Skin Erosion | 1 (1.5 %) | 5 (10.2 %) | 6 (26.1 %) | 4 (30.8 %) | 1 (25.0 %) |
| Movement of Battery | 0 | 2 (4.1 %) | 6 (26.1 %) | 2 (15.4 %) | 0 |
| Multiple Movements of Battery | 0 | 1 (2.0 %) | 1 (4.3 %) | 0 | 1 (25.0 %) |
| Surgical Revision | 1 (1.5 %) | 2 (4.1 %) | 2 (8.7 %) | 2 (15.4 %) | 1 (25.0 %) |
| Temporarily Removal of Battery | 1 (1.5 %) | 0 | 0 | 0 | 0 |
| Replacement of all Extracerebral Hardware | 0 | 0 | 0 | 1 (7.7 %) | 0 |
| Chronically Removal of DBS Equipment | 0 | 1 (2.0 %) | 0 | 0 | 1 (25.0 %) |
| Pneumonia | 0 | 0 | 1 (4.3 %) | 0 | 0 |
| Externalization | 0 | 0 | 0 | 1 (7.7 %) | 0 |

Legend: Number and Percentage of Complication or Interventions Performed due to Complications after Different Battery Replacements when the battery was changed because it ran out of power or due to dysfunction of the hardware.
Number (%)

**Supplementary Table 3.** Stimulation settings.

|  | Postoperative (N=64) | One-Year Follow-Up (N=62) | 8-15-Year Follow-Up (N=51) |
| --- | --- | --- | --- |
| Voltage [mV](D) | 2.3 [0.61] | 3.0 [0.55] | 3.2 [0.67] |
| Voltage [mV](N) | 2.2 [0.56] | 2.8 [0.57] | 3.1 [0.66] |
| Frequency [Hz](D) | 120-129: 1  130-139: 54  140-199: 7  200-210:2 | 120-129: 0  130-139: 39  140-199: 18  200-210: 6 | 120-129: 1  130-139: 28  140-199: 35  200-210: 2 |
| Frequency [Hz](N) | 120-129: 1  130-139: 55  140-199: 5  200-210: 1 | 120-129:0  130-139: 41  140-199: 18  200-210: 4 | 120-129: 1  130-139: 26  140-199: 35  200-210: 2 |
| Pulse width [ms](D) | 60: 63  90: 1 | 60: 56  90: 7 | 60: 61  90: 5 |
| Pulse width [ms](N) | 60: 63 | 60: 57  90: 6 | 60: 60  65: 1  90: 3 |
| Bilateral monopolar | 47 | 21 | 7 |
| Bilateral bipolar | 0 | 0 | 3 |
| Bilateral double monopolar | 4 | 20 | 20 |
| Bilateral triple monopolar | 0 | 1 | 1 |
| Monopolar+bipolar | 1 | 2 | 3 |
| Monopolar+double monopolar | 10 | 15 | 11 |
| Bipolar+double monopolar | 0 | 1 | 1 |
| Double monopolar+triple monopolar | 1 | 1 | 3 |
| Bilaterally turned off | 1 | 0 | 1 |
| Unilateral double monopolar and the other turned off | 0 | 1 | 0 |
| Unilateral interleaving (monopolar/bipolar) +monopolar | 0 | 0 | 1 |

Legend: Descriptive Data of Stimulation Settings at the End of the Postoperative Hospitalization, the One-Year Follow-Up, and the 8-15-Year Follow-Up (Before Reprogramming Protocol). D: Dominate Hemisphere. N: Non-Dominate Hemisphere.
Voltage: Mean [SD]
Other: Number of Patients
